# Reduction of novel circulating long-chain fatty acids in colorectal cancer patients is independent of tumor burden and correlates with age

**DOI:** 10.1186/1471-230X-10-140

**Published:** 2010-11-29

**Authors:** Shawn A Ritchie, Doug Heath, Yasuyo Yamazaki, Bryan Grimmalt, Amir Kavianpour, Kevin Krenitsky, Hoda Elshoni, Ichiro Takemasa, Masakazu Miyake, Mitsugu Sekimoto, Morito Monden, Takeshi Tomonaga, Hisahiro Matsubara, Kazuyuki Sogawa, Kazuyuki Matsushita, Fumio Nomura, Dayan B Goodenowe

**Affiliations:** 1 Phenomenome Discoveries Inc., Saskatoon, Saskatchewan, Canada; 2 Bioserve Biotechnologies, Inc., Laurel, MD, USA; 3 Saskatchewan Disease Control Lab, Regina, Saskatchewan, Canada; 4 Department of Surgery, Graduate School of Medicine, Osaka University, Osaka, Japan; 5 Department of Molecular Diagnosis, Graduate School of Medicine, Chiba University, Chiba, Japan; 6 Department of Frontier Surgery, Graduate School of Medicine, Chiba University, Chiba, Japan

## Abstract

**Background:**

Serum levels of novel hydroxy polyunsaturated ultra long-chain fatty acids (hPULCFAs) have been previously shown to be reduced in pre-treatment CRC patients compared to disease-free subjects, independent of disease stage. However, whether reduced levels of hPULCFAs result from the presence of cancer is currently unknown, as is the distribution of hPULCFAs in the general population. The following studies were carried out to assess whether conventional therapy would result in restoration of systemic hPULCFAs in CRC patients, and to investigate the relationship between hPULCFA levels and age.

**Methods:**

Tandem mass spectrometry was used to determine serum levels of the 28 carbon-containing hPULCFA C28H46O4 (CRC-446) in the following cohorts: two independent Japanese CRC populations following surgical tumor removal (n = 86), a North American Caucasian CRC cohort (n = 150) following post-surgery combination chemo/radiation therapy, 990 randomly selected anonymized serum samples from subjects ranging between 11 and 99 years of age, as well as longitudinally collected serum samples from healthy normals (n = 8, up to 90 weeks) and stage IV CRC subjects on combination therapy (n = 12, up to 63 weeks).

**Results:**

Serum CRC-446 levels in CRC subjects were significantly lower than controls (mean of 0.297 ± 0.07 ug/ml in controls versus 0.092 ± 0.03 in CRCs, p < 0.001), and were unaffected by surgical tumor removal or by chemo/radiation treatment (p > 0.05 between pre vs post surgery). CRC-446 levels showed a strong inverse association with age (p < E-11) across the randomly-selected cohort of 990 subjects, with no correlation observed in the CRC-positive subjects. Longitudinal intra-subject results, however, showed relatively stable CRC-446 levels over the short term of up to 90 weeks in both disease-free subjects and late-stage CRC patients.

**Conclusions:**

Our findings show that CRC-446 levels are not affected by conventional CRC treatment and inversely correlate with age, which suggest that reduced serum CRC-446 levels likely exist prior to the development of CRC. Extrapolation of the results to a simple screening scenario showed that, compared to fecal blood testing, pre-colonoscopy screening using serum CRC-446 levels would require 80% fewer colonoscopies, would identify risk in subjects under the age of 50, and would result in increased numbers of early cases detected. The precise role these serum metabolites play in the aetiology of cancer development remains to be determined.

## Background

Colorectal cancer (CRC) incidence, similar to many other diseases, shows a strong positive association with age. It is generally accepted that screening for colon cancer starting at age 50 should result in reduced mortality [1-4]. Many of the current screening approaches are based on either direct colonoscopic visualization of preneoplastic polyps, or on tumor-related markers such as fecal occult blood [5,6], tumor-derived transcripts [7], proteins [8-10], or methylation patterns [11], which would presumably require a threshold tumor burden before they could be to be used to positively detect cancer. Given that sporadic CRC risk is heavily influenced by factors such as age, diet, lifestyle and environment [12,13], all of which have metabolic implications, we hypothesized that there should be an underlying metabolic component associated with CRC. Using a comprehensive metabolomic approach we previously showed that serum from CRC patients exhibited reduced levels of novel 28 carbon long-chain hydroxy fatty acids compared to disease-free controls [14]. The decreased concentration of these molecules, named hydroxylated polyunsaturated ultra-long chain fatty acids (hPULCFAs), was observed in five separate case-control cohorts, was independent of ethnicity or geographic location, and showed little to no correlation with disease stage [14].

These data, combined with the structural resemblance of hPULCFAs to other endogenous long-chain fatty acids with inflammation-resolving activity [15-20], suggested to us that the observed reduction could be independent of, and possibly precede, tumor development. We herein more thoroughly investigate this hypothesis by addressing two fundamentally important questions: 1) are reduced CRC-446 levels in CRC patients the result of the tumor, and 2) do CRC-446 levels correlate with age? The findings of these studies were then used to model and discuss how a screening program based on the measurement of CRC-446 in serum could be used to identify high risk subjects, and what effect this would have on existing endoscopy resources and current CRC detection rates.

## Methods

### Patient sample selection

Samples provided by Osaka Medical University (Osaka, Japan) included 46 pre-surgery CRC patients, 34 post-surgery samples from the same pre-surgery group, and 35 controls which were prospectively collected according to the standard collection protocol of the institution. All samples were properly consented and study protocols were performed according to the ethical guidelines set by the committee of the three Ministries of the Japanese Government. Samples from Chiba University (Chiba, Japan), included 40 pre-surgery CRC patients, 40 post-surgery samples from the same subjects, and 40 controls. All samples were also consented and prospectively collected under an ethics reviewed protocol approved by the Institutional Review Board of Graduate School of Medicine, Chiba University. A summary of the populations is shown in Table 1. All samples were processed and analyzed in a randomized manner, and the results unblinded following analysis. hPULCFA discovery results including CRC-446 on the controls and pre-surgery Japanese samples were also previously reported [14]. Serum samples for the age-association study were provided as anonymized reference samples from the Saskatchewan Disease Control Lab (SDCL, Regina, Saskatchewan, Canada) in accordance with SDCL policies and under a protocol approved by the Ethics Department of the University of Saskatchewan. Serum samples for longitudinal analysis in CRC-free subjects were properly consented and collected according to standard operating procedures at Phreedom Santé, Inc., Saskatoon, Saskatchewan, Canada. Longitudinal samples from stage IV metastatic subjects were properly consented and collected under an ethically approved clinical trial protocol by Biomira Inc., Edmonton, Alberta, Canada. Serum samples from North American Caucasian CRC patients were collected, processed and stored in a consistent manner by teams of physicians as part of a global initiative using standardized protocols and operating procedures at BioServe Biotechnologies, Inc., Laurel, Maryland, USA. Collection protocols were approved by the Western Institutional Review Board, and all samples were properly consented. All samples were accompanied by detailed pathology reports which were independently verified by certified pathologists.

**Table 1:** Summary of clinical populations.

	SDCL	Longitudinal 1	Longitudinal 2	North American	Osaka		Chiba	
**Disease status**	Controls	Controls	CRC	CRC	CRC^1^	Control^1^	CRC^1^	Control^1^

**Total Subjects**	990	8 (110 samples)	12 (94 samples)	150	46	35	40	40

**Ethnicity**	Caucasian	Caucasian	Caucasian	Caucasian	Japanese	Japanese	Japanese	Japanese

**< age 40**	214			6				
**age 40-49**	175			11				
**age 50-59**	189			33				
**age 60-69**	164			47				
**age 70-79**	133			34				
**age >80**	116			19				

**Male N**	320	4		75	27	na	19	24
**Male Age Range (yrs)**	6-93	36-61		66 (33-91)	63 (28-90)	na	68 (45-91)	48 (36-69)
**Female N**	670	4		75	19	na	21	16
**Female Age Range (yrs)**	11-99	33-44		63 (31-89)	65 (31-77)	na	70 (51-84)	49 (39-59)

**Stage 0/I**				12	10		9	
**Stage II**				30	14		18	
**Stage III**				46	12		11	
**Stage IV**				42	8		2	
**Unknown**				20	2		0	

**Total surgery**				139	34^2^		40^2^	
**Chemotherapy**^**3**^				76	na		na	
**Chemo/Radiation**^**3**^				31	na		na	
**No Chemo/Radiation**^**3**^				37	na		na	
**No Surgery**				11	na		na	

^1^Control and pre-treatment samples are the same ones reported in [11].

^2^Not previously reported.

^3^Therapy following surgery.

### Sample extraction and mass spectrometry analysis

Serum samples were stored at -80°C until thawed for analysis, and were only thawed once. All extractions were performed on ice as previously described [14]. Briefly, 10 ug/ml [^13^C_1_] cholic acid was added to the serum prior to extraction and the samples sequentially extracting using equal volumes of serum with 1% ammonium hydroxide and ethyl acetate (EtOAc) four times. Samples were centrifuged between extractions at 4°C for 10 min at 3500 rpm, and the organic layer was transferred and pooled to a new tube (extract A) for each sample. 100 uL of the extract A were injected by flow-injection analysis into the 4000QTRAP™ equipped with a TurboV™ source with an APCI probe as previously described [14]. The method is based on the multiple reaction monitoring (MRM) of one parent ion transition for hPULCFA C28H46O4 (CRC-446, 445.3/383.4 [M-H] Da), and a single transition for the internal standard (408.3/343.4 [M-H] Da). Metabolite concentrations represented as [^13^C_1_] cholic acid equivalents (CAEs) were then extrapolated, normalized by dividing by the percent recovery, and multiplied by appropriate extraction dilution factor to yield a final serum concentration equivalent.

### Statistical analysis

Tandem mass spectrometry data was analysed using JMP version 8.0.1 and Microsoft Excel. Two-tailed unpaired Student's *t*-tests were used for determination of significance between comparison groups. *P*-values of less than 0.05 were considered significant. Receiver-operator characteristic (ROC) curves were generated using JROCFIT 1.0.2 (http://www.jrocfit.org).

## Results

### CRC-446 levels following surgery

**Figure 1:**
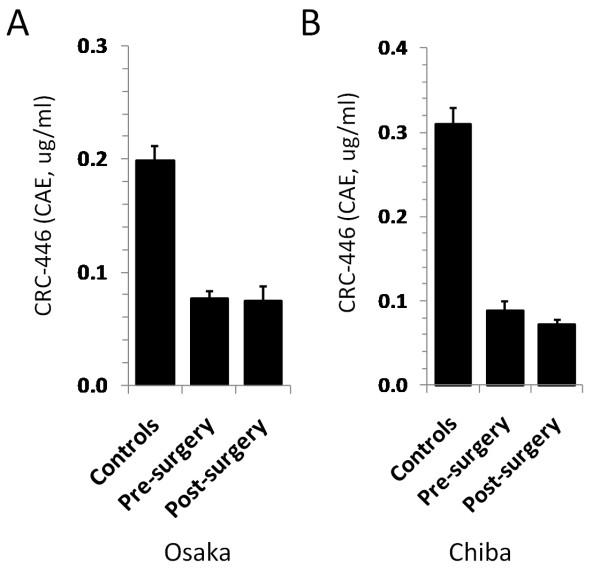
**CRC-446 levels in pre and post-surgery CRC patients**. Mean levels of CRC-446 in controls, pre-surgery and post-surgery CRC patients as determined by triple-quadrupole tandem-MS for the Osaka (A) and Chiba (B) studies. Levels are reported as concentration equivalents (± 1 S.E.M in ug/ml), to the internal standard [^13^C_1_]cholic acid, (CAEs). *p*-values based on Student's t-test between pre- and post-surgery in both studies were >0.05. Note: Control and pre-surgery levels were also previously reported in [14].

To investigate whether reduced CRC-446 levels in CRC patients were the result of tumor burden (i.e., does the tumor itself utilize or metabolize hPULCFAs), levels in CRC patients prior to and following surgery were determined to see if tumor removal would result in CRC-446 restoration. Serum levels of CRC-446 were assayed using tandem mass spectrometry (and reported as cholic acid equivalents; see materials and methods) for samples from two separate cohorts of CRC patients prior to and following surgical tumor removal as well as matched controls. Consistent with previous findings [14], the CRC patient pretreatment CRC-446 levels in both the Osaka and Chiba studies showed significantly lower (p < 0.001) levels than their respective control groups. The mean CRC-446 level in the Osaka study control group was 0.20 ± 0.014 ug/ml CAEs while the CRC pre-surgery mean was 0.077 ± 0.006 ug/ml CAEs. The Chiba CRC-446 control mean was 0.31 ± 0.02 ug/ml CAEs and the presurgery mean was 0.089 ± 0.006 ug/ml CAEs. In both patient cohorts (34 post-surgery from Osaka, and 40 post-surgery from Chiba), CRC patients showed statistically similar mean CRC-446 levels before and after surgery (Figures 1A and 1B respectively, p > 0.05 for pre-versus post-surgery in both studies). The mean CRC-446 level following surgery in the Osaka study was 0.075 ± 0.012 ug/ml CAEs and in the Chiba post-surgery group it was 0.072 ± 0.006 ug/ml CAEs. For both studies, the means of the differences between the pre- and post-surgery CRC-446 levels were zero, and there was no correlation between CRC-446 level and time of collection following surgery (up to 377 days after surgery; not shown). The results showed that surgical removal of CRC tumors in patients did not result in the restoration of CRC-446 levels.

### CRC-446 levels following combination surgery/chemo/radiation therapy

**Figure 2:**
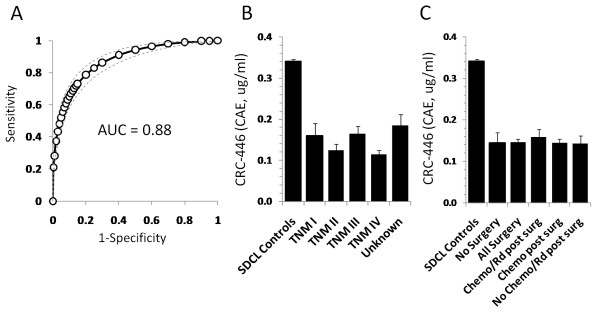
**CRC-446 levels in controls and CRC patients**. (A) ROC analysis based on CRC-446 concentrations across 150 Caucasian post-treatment CRC patients and 761 age-matched controls. Dotted lines represent the 95% confidence interval. Mean CRC-446 levels (± 1S.E.M) are shown by disease stage for the 150 CRC patients (B) and by treatment combination (C). *p*-values based on Student's t-test between all stages and between treatment comparisons were >0.05.

To further investigate possible effects of tumor burden, CRC-446 levels were measured in 150 North American Caucasian CRC patients following various combinations of chemo and radiation therapy following surgery (Table 1). For reference, we also included results from a subset of 761 age-matched North American subjects from the SDCL general population described below, which showed a reference mean CRC-446 concentration of 0.33 ± 0.005 ug/ml CAEs. Relatively low CRC-446 levels were observed across the post-treatment CRC-positive cohort, with a mean level of 0.15 ± 0.008 ug/ml CAEs. The corresponding receiver-operator characteristic (ROC) curve is shown in Figure 2A, which resulted in an area under the curve (AUC) of 0.88. No correlation between CRC-446 levels and disease stage was observed (all stage comparisons p > 0.05, Figure 2B). These levels were consistent with previously reported North American Caucasian cohort data where pre-treatment CRC levels were reported to be approximately 0.13 ug/ml (CAEs), with an ROC AUC of 0.876 [14]. In the current North American post-treatment CRC cohort, we observed no correlation between CRC-446 levels and gender, age or BMI (all p > 0.05; not shown). Comparison of five subsets of treatment groups within the patient cohort including those who had surgery, those who did not have surgery, those of the patients that did have surgery who subsequently had chemo, combination chemo/radiation, or no chemo/radiation, revealed no significant differences in the levels of CRC-446 between any of the treatment combinations (Figure 2C; p > 0.05 for all comparisons). The results were consistent with previous data showing a lack of correlation between CRC-446 levels and disease stage, that low CRC-446 levels did not result from the presence of a colorectal tumor, and that CRC-446 levels were unaffected by any form of treatment.

### Effect of age on circulating levels of CRC-446 in control subjects and CRC patients

**Figure 3:**
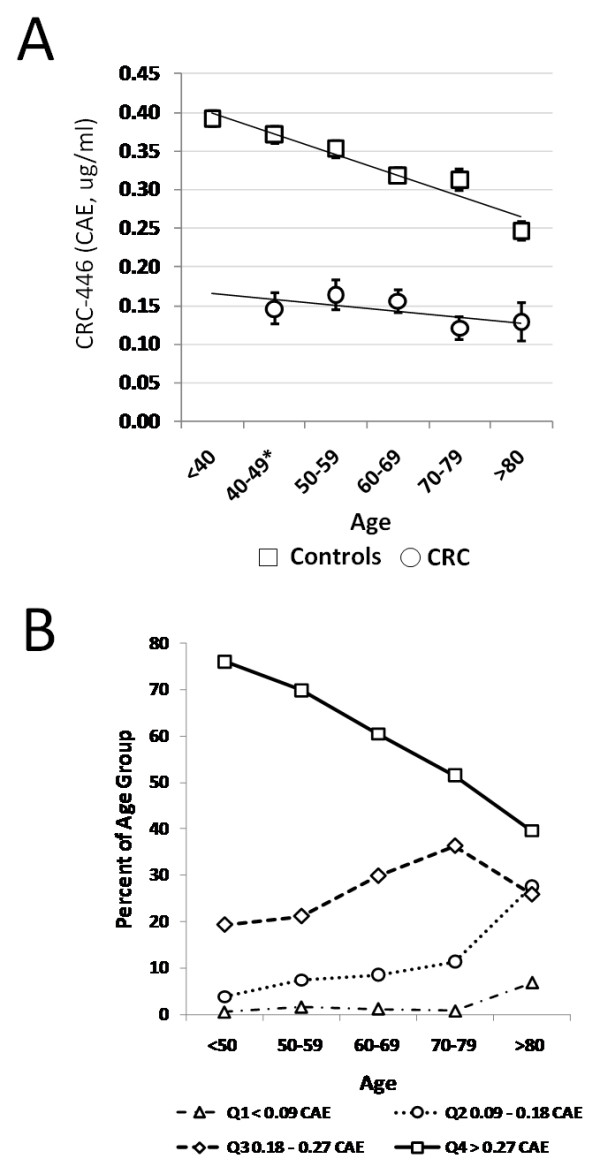
**CRC-446 levels in controls and CRC patients by age**. (A) Mean CRC-446 levels (± 1 S.E.M.) by decade of life for the 990 controls (open squares) and for 150 CRC post-treatment patients (open circles). (B) Percent of subjects by age group falling into four CRC-446 quartiles centered around 50% of the under age 50 mean concentration of 0.18 ug/ml CAE.

The lack of correlation between serum CRC-446 levels and tumor burden, in combination with the age-associated increase in CRC incidence, prompted us to question the possibility of a correlation between age and CRC-446 levels by assaying a random population of 990 control subjects aged 11-99 collected at the provincial clinical testing lab in Regina, Saskatchewan, Canada (SDCL, Table 1). A significant inverse correlation between the concentration of CRC-446 and age was observed in both the male and female cohorts. Regression analysis (by gender) between CRC-446 levels and age resulted in R values of 0.26 and 0.38, F-values of 48 and 55, and p-values of 8.4E-12 and 1.2E-12 for males and females, respectively (Table 2). Similar analysis in the North American post-treatment CRC patient population revealed no significant correlation (regression R value of 0.11, F-value of 1.8 and p-value of 0.18; Table 2). The ten-year average decline (± 1 S.E.M) in CRC-446 with age in the general population relative to CRC positive subjects is shown in Figure 3A.

**Table 2:** Regression analysis between CRC-446 levels and age

Cohort	Multiple R	F	P-value
SDCL female controls	0.26	48	8.4E-12

SDCL male controls	0.38	55	1.2E-12

Bioserve CRC patients	0.11	1.8	0.18

To investigate this age-related phenomenon further, we defined the "optimal" CRC-446 level as greater than 50% of the mean concentration of subjects under age 50 (>0.18 ug/ml CAEs). The percent of subjects by decade of life falling into four quartiles centered around this concentration (ie, <0.09, 0.09-0.18, 0.18-0.27 and >0.27 ug/ml CAEs) were then determined and plotted as shown in Figure 3B. A remarkable decline in the percentage of subjects at each decade of life in the highest CRC-446 quartile is observed, with an increase in the percent of subjects per decade of life in the middle two quartiles. Relatively few subjects were present in the lowest quartile. Specifically, the data indicate that 75% of the under 50 age group have CRC-446 levels that are in the top quartile (>0.27 ug/ml CAEs) while approximately 20% have levels in the second highest quartile (0.18-0.27 ug/ml CAEs) and that approximately 5% have levels in the bottom two quartiles (<0.18 ug/ml CAEs). By age 70-79, the percent of subjects in the highest quartile drops to nearly 50%, with over 35% of subjects in the second highest quartile and over 10% in the lowest two quartiles (Figure 3B). Interestingly, the sum of the lowest three quartiles shows a nearly perfect inverse correlation with the top quartile (correlation coefficient of -0.999, not shown). The implications of these findings are expanded upon in the final sections of the results and discussion.

### Longitudinal CRC-446 stability

**Figure 4:**
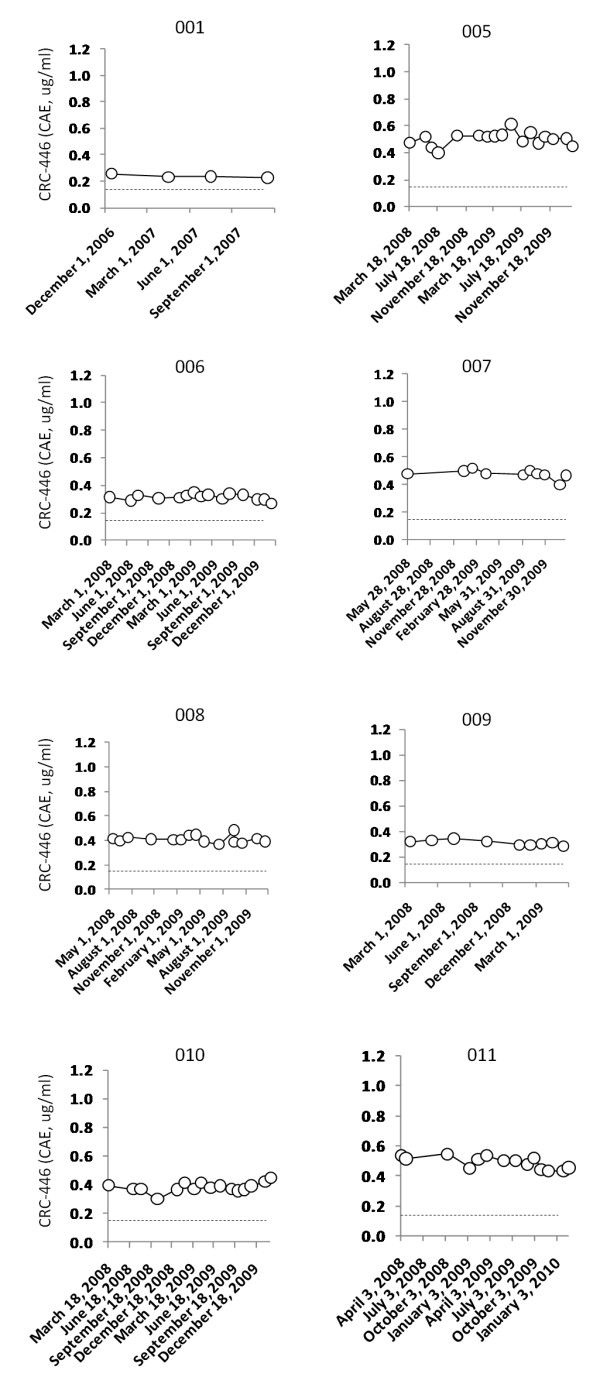
**Longitudinal CRC-446 levels in controls**. Levels of CRC-446 at multiple timepoints up to 90 weeks are shown for eight healthy asymptomatic subjects. The dotted lines represent the mean CRC-446 concentration in CRC-positive subjects of 0.15 ug/ml CAEs for reference.

**Figure 5:**
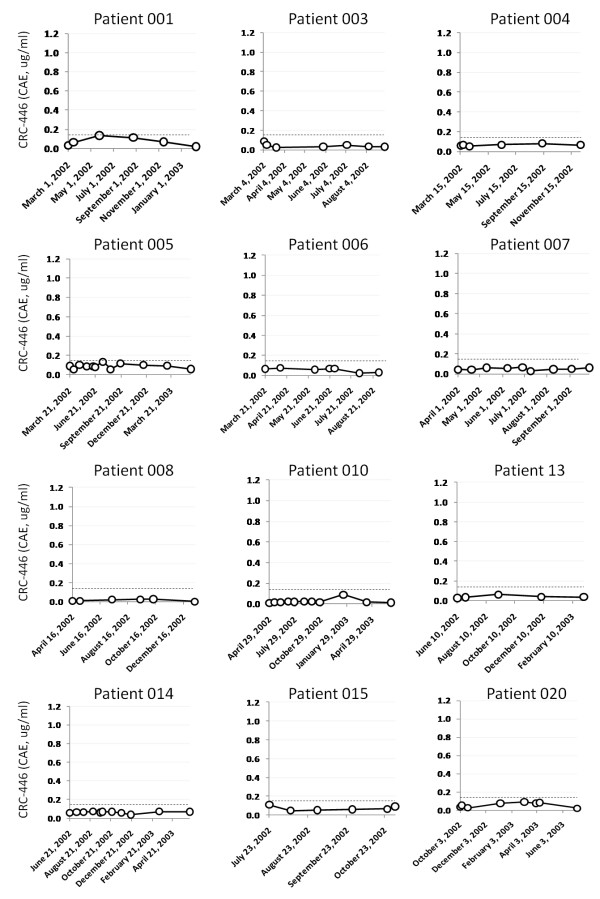
**Longitudinal CRC-446 levels in stage IV metastatic CRC patients**. Levels of CRC-446 at multiple timepoints up to 63 weeks are shown for 12 stage IV metastatic CRC patients enrolled in a clinical trial for an experimental cancer vaccine. The y-axes are scaled equivalently to Figure 4 for comparison and the dotted lines represent the mean CRC-446 concentration in CRC-positive subjects of 0.15 ug/ml CAEs for reference.

Next we investigated the short-term intra-individual variability of CRC-446. Eight healthy volunteers (four females and four males, Table 1) were sampled frequently over an approximate 90 week period. CRC-446 in these subjects showed high intra-individual stability (Figure 4), with an average percent CV of 7.6 ± 1.2% across the eight subjects. All samplings from the eight subjects were considerably higher than the average CRC patient levels (above 0.15 ug/ml CAEs, dotted lines in Figure 4). A similar analysis was performed longitudinally for up to 63 weeks on 12 stage IV CRC patients enrolled in a phase IIb clinical trial for an experimental vaccine (Figure 5). Although the intra-individual stability was higher in CRC patients (average percent CV of 41.8 ± 22.3%), the relative CRC-446 levels were very low (average of 0.055 ± 0.003, ug/ml). The higher variability was likely due to the very low CRC-446 signal-to-noise levels which were in the lower detection range of the mass spectrometer. As expected, in both the control and CRC longitudinal cohorts, there were no statistically significant intra-individual trends with time (all *p *for regression > 0.05). The results show that in the short term, intra-individual CRC-446 levels are relatively stable and resistant to acute lifestyle factors such as diet. Collectively the results indicate that CRC-446 levels do not randomly increase or decrease in the population over time, but that either population CRC-446 levels decline slowly over a relatively long period of time or CRC-446 levels decline rapidly due to some unknown acute event in a subset of subjects and then remain stably low in these subjects for long periods of time.

### Impact of an age-associated biomarker on screening

**Figure 6:**
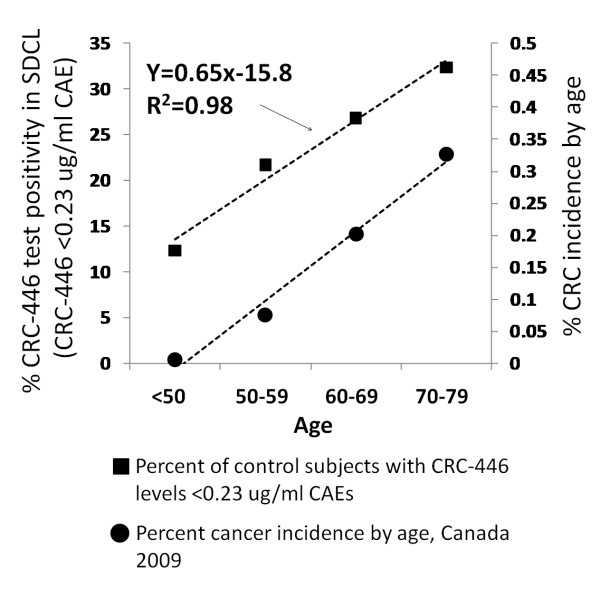
**Age-related CRC-446 positivity and CRC incidence rates**. Percent of SDCL control subjects by decade of life with CRC-446 levels less than 0.23 ug/ml CAE (squares, primary y-axis). Linear regression analysis (based on the median age of each decade of life for the SDCL group) was used to determine the equation y = 0.65x-15.8, representing a .65% annual probability of a positive test result (concentration less than 0.23 ug/ml CAE) given a prior negative test (concentration greater than 0.23 ug/ml CAE). For comparison, the incidence rate of CRC as a percent of each age group (based on Canadian Cancer Statistics, 2009), is shown (circles, secondary axis).

The above results suggest that the increased prevalence of low CRC-446 levels with age is a cumulative process. This phenomenon creates a unique screening situation in that, unlike other screening tests, the CRC-446 level of a subject is influenced by both the previous test result and the inter-test interval. For example, using a 0.23 ug/ml CAE cut-off (selected to give an age-adjusted positivity rate of approximately 10% in the SDCL control population aged 50 or less) the relationship between the cumulative positivity rate, based on this cut-off, and age for subjects aged <50, 50-59, 60-69 and 70-79 is linear (Y = 0.65X-15.8, R2 = 0.98, Figure 6). The over 80 age group was excluded from the model since screening subjects of this age has questionable benefit [21]. The relationship between the increase in percent of subjects with CRC-446 levels below 0.23 ug/ml CAEs by decade of life and the increase in percent CRC incidence across the same age groups is further shown in Figure 6. The slope of the line for CRC-446 levels by age was determined through linear regression to be 0.65 (with an R^2 ^of 0.98), corresponding to an annual increase in the probability of having a CRC-446 level drop below 0.23 ug/ml CAEs of approximately 0.65% (or a 10-yr cumulative probability of approximately 6.5%) given a prior negative test. The implications of these findings on colonoscopy resources and CRC detection rates during the projected lifetime CRC screening guidelines of the general population are discussed below.

## Discussion

Age is the most robust risk factor for the majority of human diseases including cancer (Centers for Disease Control and Prevention (http://www.cdc.gov 2010)). Since most diseases likely have a long prodromal phase, it is reasonable to postulate that causative biochemical perturbations would begin to appear well before the clinical onset of symptoms. Our previous findings that the hPULCFA reduction in CRC patients was independent of disease stage [14] prompted us to further investigate whether this reduction was also independent of tumor burden, and if so, whether there was any correlation between hPULCFA levels and age given the age association with CRC incidence rates. The lack of CRC-446 restoration in two independent cohorts of CRC patients following surgery, and indistinguishable levels between pre- and post-combination therapy reported herein show that the reduction of circulating hPULCFA levels in CRC patients is not due to the presence of cancer. A cross-sectional analysis of the general population revealed a strong inverse association between age and CRC-446 levels in average risk subjects but no age correlation in CRC subjects. Longitudinal analysis in healthy normals and late stage CRC subjects revealed that within-subject CRC-446 levels are stable for at least 1 year. Together, these data support the conclusion that hPULCFA levels decline prior to CRC development and that the percentage of the population that exhibits this decline increases with age. The cause of this decline is unknown.

The above characteristics differentiate CRC-446 from other CRC markers as many reported markers are tumor-derived (i.e., fecal blood, transcripts, proteins, methylated DNA, etc.). To investigate the screening implications based on using CRC-446 for identifying elevated CRC risk, we modelled the predicted outcomes of a hypothetical cohort of 100,000 subjects living from age 30 to 80, and compared the impact on colonoscopy resources and detection rates to that of fecal immunological testing (FIT) (Table 3). The equation y = 0.65x-15.8, generated from CRC-446 data reported in this paper, was used to estimate per-decade age-specific positivity rates for a typical screening population over a 50-year timeframe. We set a threshold that resulted in a 10% positivity rate in subjects under the age of 50 (0.23 ug/ml CAE), which is comparable to the Fecal Immunochemical Test (FIT) [22]. Since the percent probability of having an annual subsequent positive CRC-446 test result when the first test was negative is only 0.65%, the expected per-decade positivity rate (following an initial negative test) would be 6.5%. Interestingly, using a threshold this high resulted in an average test sensitivity of 83.5% when applied to the BioServe post-treatment CRC cohort (Table 3). Extrapolation of these positivity rates to a theoretical biennial average risk screening cohort of 100,000 would therefore result in approximately 30,000 positive CRC-446 test results and subsequent recommended follow-up colonoscopies. In comparison (and everything else being equal), biennial FIT testing would require 150,000 recommended follow up colonoscopies (assuming a 10% positivity rate, Table 3) and would exclude subjects under age 50 based on current guidelines. In fact, if the CRC-446 threshold was raised to a point which resulted in 150,000 positive CRC tests and subsequent follow up colonoscopies (five times higher than the current model and equal to that of FIT), the sensitivity of CRC-446 would be well over 90%. It is critical to point out that a key difference between the CRC-446 marker and a non age-related biomarker such as FIT for screening is that increasing or decreasing the frequency of CRC-446 tests (for example, to one every five years rather than one every two years as modelled) would *not *result in more follow up colonoscopies required since the probability of a subsequent positive test is simply the interval time × 0.65, whereas the 10% positivity for FIT is independent of the interval time.

**Table 3:** Comparison of test positivity rates and recommended follow-up procedures based on CRC-446 (<0.23 ug/ml CAEs) and FIT.

Age	Actual CRC-446 test positivity (% by decade) for SDCL controls (<0.23 ug/ml)	**Theoretical CRC-446 positivity (incremental % by decade**^^**1**^^**) for SDCL controls (y = 0.65x-15.8)**	Actual Bioserve CRC positivity (% by decade using <0.23 ug/ml)	**Estimated follow-up colonoscopies per decade based on CRC-446 test**^^**3**^^	**Estimated follow-up colonoscopies per decade based on biennial FIT**^^**4**^^	**Expected incidence from initial 100,000**^^**6**^^	**CRC-446 test estimated CRC detections**^^**7**^^	**FIT test estimated detections**^^**8**^^
<50	12.3	10^**2**^	88.2	10,000	0	250	220	132
50-59	21.7	6.5	81.8	6,500	50,000	695	569	417
60-69	26.8	6.5	78.7	6,500	50,000	1684	1326	1010
70-79	32.3	6.5	85.3	6,500	50,000	2824	2409	1694

(Average) Total			**(83.5)**	**30,000**	**150,000**	**5453**	**4523**	**3254**

^1^Based on equation y = 0.65x-15.8 fitted to SDCL control data per decade falling below CRC-446 levels of 0.23 ug/ml.

^2^First CRC-446 test positivity (adjusted to mean of age 30 to 50).

^3^Note that number of positive CRC-446 tests is independent of testing frequency. Values shown are for per decade cumulative risk and assume all CRC-446 positive subjects have colonoscopy.

^4^Based on performing one FIT test every two years with 10% positivity rate (assuming all FIT positive subjects have colonoscopy).

^6^Based on the probability of developing cancer by decade of life (2009 Canadian Cancer Stats).

^7^Based on reported Bioserve CRC decade-specific positivity rates.

^8^Based on an average sensitivity of 60% per decade.

The impact on CRC detection rates was next estimated across the 100,000 person cohort based on the reported probability of developing CRC in the next decade of life (Canadian Cancer Statistics, 2009). Using the decade-specific sensitivity rates reported for the BioServe population in this paper, approximately 4525 of the estimated 5453 cases (83%) of the CRC cases would be expected to be detected, while FIT (based on an age-independent sensitivity of 60% per decade, but which can vary widely depending on the study [23]), would result in approximately 3254 positive detections. The key points to these observations are: 1) the implementation of CRC-446 as a screening biomarker for CRC would require significantly fewer follow-up colonoscopies than FIT, 2) more CRC cases would likely be detected, 3) CRC cases under the age of 50 could be detected which are currently missed, and 4) since there is no bias between CRC-446 levels and disease stage, there would be an enrichment in the number of early stage cancers detected. The impact in terms of cost-benefit could be substantial. For example, assuming similar frequency and cost of CRC-446 versus FIT and a colonoscopy cost of $1000, the use of CRC-446 would result in approximately $120 M in reduced follow-up colonoscopy screening costs per 100,000 subjects. In addition, the enrichment of early-stage cancers would also translate into reduced healthcare burden simply due to the lower direct cost of treating early-versus late-stage cancer, as well as the indirect cost savings associated with decreased mortality. However, detailed cost-benefit analyses for individual geographic regions would be required to accurately forecast the economic health-burden impact of CRC-446 screening. Unfortunately, the lack of correlation between CRC-446 levels and any form of treatment suggests that CRC-446 would likely not be suitable for monitoring treatment response. However, whether CRC patients with "normal" CRC-446 levels (i.e., false negatives) have a different prognostic outcome than CRC subjects with low CRC-446 levels remains to be determined.

Although the origin of hPULCFAs, including CRC-446, is currently unknown, they are structurally similar to the fatty acid-derived long-chain hydroxylated resolvins [24-26] and protectins [27-29], which are mediators involved in the resolution of acute inflammation. The failure to resolve acute inflammation can lead to a chronic inflammatory state, a well known underlying component of CRC development [30] which is the basis for chronic low-dose preventative NSAID use [31,32]. The possibility that CRC-446, or other hPULCFAs may be to some extent implicated in mediating endogenous anti-inflammatory responses cannot be excluded at this time, and further studies will be required to investigate any role(s) that these molecules may play in inflammation.

There are several limitations to the results reported here. The age-related decline is based upon single point cross-sectional data, and the longitudinal studies were insufficiently powered to accurately determine the long-term rate of intra-individual decline in the general population. The design was sufficient to determine short-term intra-subject CRC-446 stability but not long-term CRC-446 instability, and thus we were unable to distinguish between a slow decline process versus a rapid decline followed by subsequent long-term stability. Ultimately longitudinal tracking of thousands of subjects over several decades will be required to determine intra-individual rates of hPULCFA decline. This age-related phenomenon is not observed for other CRC screening modalities including fecal blood, which shows no correlation between age and test positivity [33].

The fact that reduction of hPULCFAs in CRC patients does not correlate with disease stage, that the reduction is not affected by surgery or treatment, and that an average population shows an age-related reduction which correlates with the incidence of the disease, suggests that the reduction of hPULCFAs occurs prior to and is independent of the presence of the cancer. Such a disease risk marker is indeed unique in the CRC field.

## Conclusions

The results presented herein confirm our previous report that low circulating levels of CRC-446 is a sensitive serum biomarker for CRC presence and that the low CRC-446 levels observed in CRC subjects are independent of tumor burden or the subject's age. In a representative non-CRC population, declining CRC-446 levels were observed to exhibit a strong age-association. CRC-446 reduction in non-CRC subjects, therefore, may represent an elevated risk for subsequent CRC development. Due to the sensitivity and the cumulative nature of the observed CRC-446 deficiency, a CRC screening program based upon serum CRC-446 depletion is projected to detect more CRC subjects with fewer colonoscopies required per subject screened. Further studies to unambiguously demonstrate a causal relationship between reduced circulating hPULCFA levels and CRC are warranted.

## List of abbreviations

hPULCFA: hydroxy polyunsaturated ultra long-chain fatty acid; CRC: colorectal cancer; SDCL: Saskatchewan Disease Control Lab; EtOAc: ethyl acetate; MRM: multiple reaction monitoring; CAE: cholic acid equivalent; ROC: receiver-operator characteristic; BMI: body mass index; AUC: area under the curve; FIT: fecal immunological test.

## Competing interests

The following authors are or were full-time employees of Phenomenome Discoveries Inc. (PDI) when this work was carried out: Shawn A Ritchie, Doug Heath, Yasuyo Yamazaki, Bryan Grimmalt, Amir Kavianpour and Dayan B Goodenowe. Only DBG (President and CEO) owns shares in PDI. Kevin Krenitsky, Ichiro Takemasa, Masakazu Miyake, Mitsugu Sekimoto, Morito Monden, Takeshi Tomonaga, Hisahiro Matsubara, Kazuyuki Sogawa, Kazuyuki Matsushita, and Fumio Nomura have no competing financial interests. PDI is financing the article processing fees for the manuscript. SAR and DBG are named inventors on patents relating to the use of CRC-446 for CRC screening, and have both received salaries from PDI, the organization named in the patents.

## Authors' contributions

SAR, lead author and writer, analyzed data including statistical analysis, data interpretation, DH, development of original hPULCFA triple-quadrupole MSMS methods, YY, Japanese study designs and data intepretation, BG, optimization and QAQC of CRC-446 test method, AK, optimization of CRC-446 test and MS analysis of Bioserve and SDCL samples, KK, design of collection protocols and study design for the Bioserve post-treatment CRC patient cohort including reviews of clinical data and pathology, HE, responsible for SDCL control study design, determination of samples appropriate for the required distributions, selection and coordination of all samples, and analysis of results, IT, design of Osaka trial, patient recruitment and confirmation of pathological findings, MM, clinical trialk design, clinical staging, and post-surgery data collection and follow up. MS, Head of the lower gastroenterological surgery group, and responsible for trial design and protocol submissions, MM, group leader at Osaka Department of Surgery involved in experimental trial design, data analysis and unblinding of patient data, TT was involved in the clinical trial design of clinical trials in Chiba, patient selection and pre-versus post surgery data collection and analysis, HM, Head of Surgery at Chiba responsible for patient selection and enrolment, KS, collection of clinical samples and clinical patient data, KM was involved in the clinical trial design of clinical trials in Chiba, patient selection and responsible for patient recruitment and trial design, FN, Project and group leader at Chiba, responsible for protocol approvals, enrolment criteria and unblinding of data, DBG, leader for work at Phenomenome Discoveries, including method development, interpretation of data, and overall direction of the manuscript. All authors have read and approved the final manuscript.

## Pre-publication history

The pre-publication history for this paper can be accessed here:

http://www.biomedcentral.com/1471-230X/10/140/prepub
